# Supplementary material for: Nanoscale Confinement Enhances Ultrafast Demagnetization
Source: Adv Sci (Weinh). 2026 Aug 12:e77058. Online ahead of print. doi: 10.1002/advs.77058 (PMC13464455; doi:10.1002/advs.77058)
Supplement: Supplementary file 1 [file ADVS-9999-e77058-s001.pdf]

Supplementary Information

for

"Nanoscale Confinement Enhances Ultrafast Demagnetization Amplitudes"

1. Magnetic characterization 1
2. Analysis of the lattice temperature from UED-results 2
3. Excitation calculation: 3
4. Data omitted for clarity 3
5. Phenomenological Simulations 4
6. References 6

1. Magnetic characterization

To determine the magnetic saturation field of the investigated samples, transmission of circularly polarized X-rays was measured at the Fe L_3 edge as a function of the externally applied magnetic field (without laser excitation). The field-dependent contribution to the transmitted intensity is dominated by the XMCD effect, and is proportional to the magnetization. The field was applied in the same orientation as in the main text (60 degrees to the plane). Fig. S1 presents the hysteresis curves measured on the 16 nm and the 8 nm samples. Based on this we used $\pm 0.4\ T$ fields in the experiment to ensure saturation.

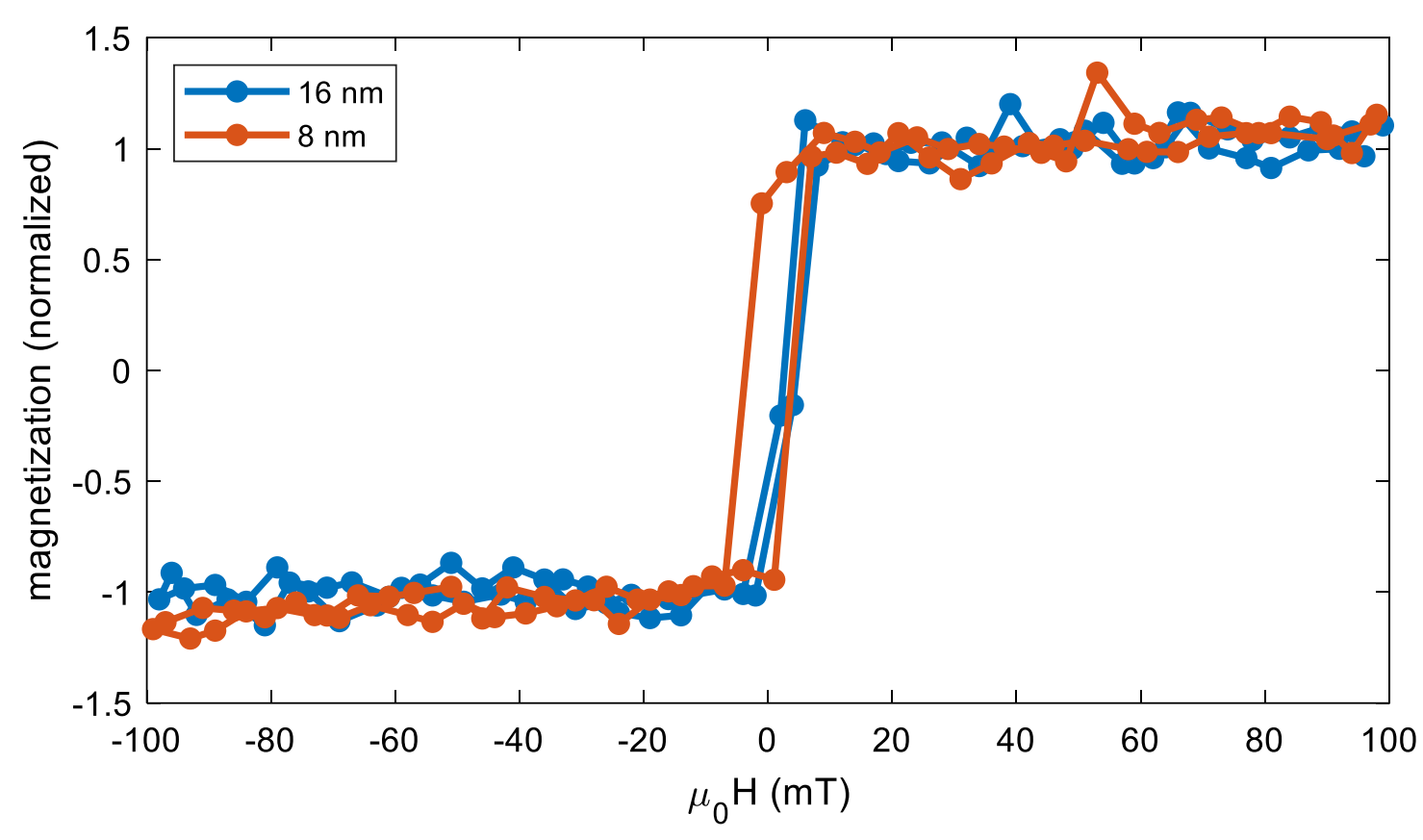

Fig. S1 – Hysteresis curves measured using XMCD in transmission. The curves were taken in the same experiment and in the same orientation as the data in the main text. For clarity, the data were normalized and their baseline was removed. The apparent exchange bias in the 8 nm sample is likely due to this being a virgin curve (sample was not perfectly saturated before the scan).

2. Analysis of the lattice temperature from UED-results

As described in the main text, the weakening of Bragg peaks (Fig. 3c) is due to increased lattice vibrations in Fe. The change in the Fe MSD ($\Delta\langle u^2\rangle$, see Fig. 3d) can be extracted from any peak extracted using Eq. M3. However, the heterostructures samples in this work consist of Fe and MgO. The lattices of these materials produce Debye-Scherrer rings at nearly identical q. In combination with the finite momentum resolution of the UED experiment, most diffraction peaks visible in our data represent a superposition of neighboring peaks from MgO and Fe. Therefore, for determining the actual $\Delta\langle u^2\rangle$ of Fe alone, we used two Fe peaks, (211) and (321), for which the neighboring MgO-peaks are insignificant.

To illustrate this, Fig. S2a presents $\Delta\langle u^2\rangle$ values extracted using Eq. M3 from these two peaks, as well as a third Fe peak, (110) at 3.1 Å^{-1}, for which the MgO contribution at 2.9 Å^{-1} is not negligible. The transient $\Delta\langle u^2\rangle$ curves from the (211) and (321) peaks are in excellent agreement with each other, while the curve from (110) exhibits a reduced amplitude, because the MgO contribution does not experience a change in MSD due to the pump pulse. The excellent quantitative agreement between the curves from (211) and (321) indicated that these reliably represent the behavior of Fe alone.

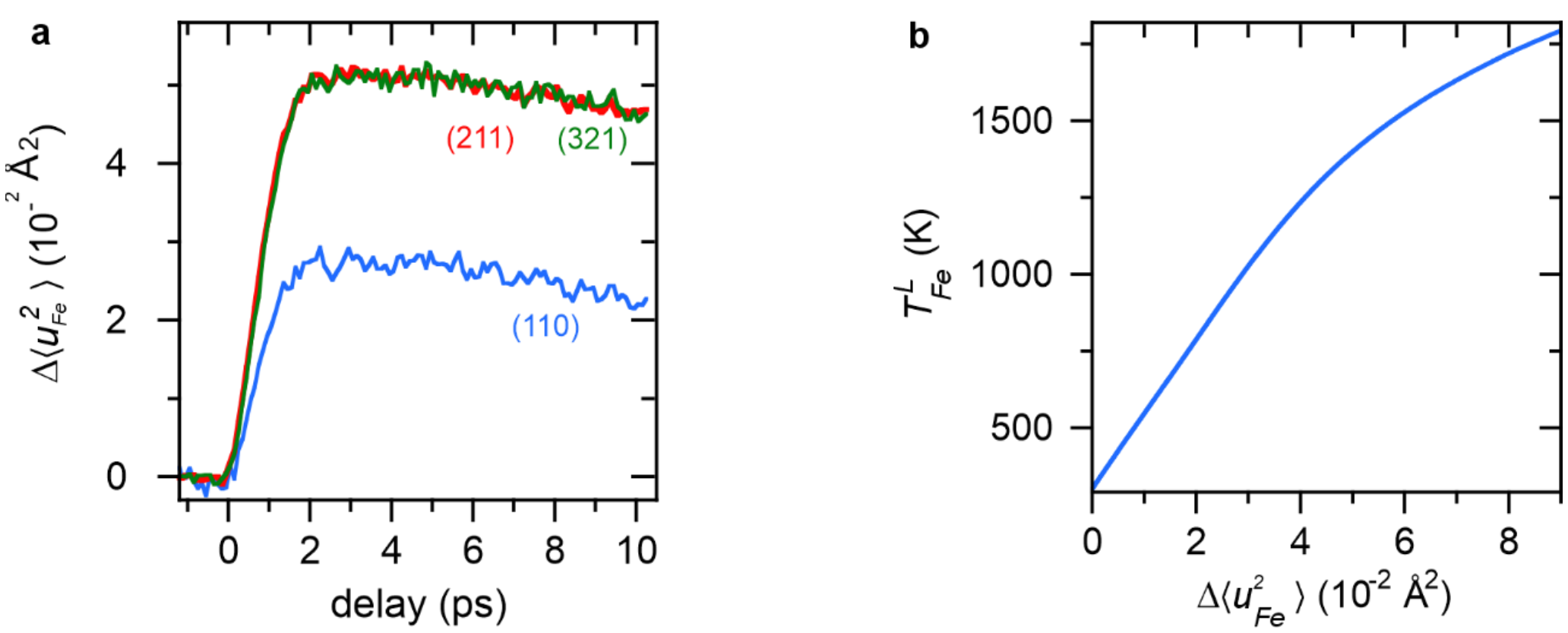

Fig. S2 – **a** Change in Fe MSD, as extracted from different Bragg peaks using Eq. M3, as a function of pump-probe delay. The data are taken from the Fe/MgO bilayer (see main text), excited at a fluence of 9 mJ/cm^2. The labels represent the Miller indices of the Fe peaks used to produce each curve. The (110) exhibits a reduced amplitude, indicating that this peak's intensity is contaminated by intensity from MgO peaks. **b** Lattice temperature of Fe as function of the change in MSD above 300K (see methods section).

The Change in Fe MSD was converted into units of temperature, as described in the "methods" section. Fig. S2b present the relation between the change in Fe MSD and the lattice temperature based on this conversion. There are two aspects that need to be considered with respect to this conversion of the measured change in MSD into a transient lattice temperature in Fe. First, significant data scatter is apparent in published temperature dependent Debye-Waller factors for Fe (see Kharoo et al.[1] and Singh et al.[2], and references therein), particularly at high temperatures. This doesn't affect our conclusions but suggests that absolutes temperature values should be treated with caution. Second, by converting MSDs into temperatures, we implicitly assume a thermal phonon population, which might not be the case for all delay times. In fact, recent experimental work has shown that even in single-element metals like Au[3], Ni[4], and Sb[5], the phonon population exhibits a non-thermal distribution for times up to a few ps. However, recent first-principles calculations of nonequilibrium mode-resolved phonon distributions[6] showed that a mode-averaged temperature is very well represented by the lattice temperature obtained from a simple two-temperature model.

3. Excitation calculation:

For the X-ray transmission experiments, the excitation density was calculated for each sample as follows. Using the incident fluence f and the photon energy E, the incoming photon flux per pulse is

$$N_{in} = f/E \;\; [ph/cm^2]$$

Using the sample thickness T and the absorption α, the transmitted and absorbed photon flux are

$$N_{trans} = N_{in}\exp(-\alpha T) \;\; [ph/cm^2]$$

$$N_{abs} = N_{in} - N_{trans} \;\; [ph/cm^2]$$

The excitation density per unit cell was then approximated using the unit cell volume V as

$$ED \approx N_{abs}/T/V \;\; [ph/UC]$$

The absorption used was 4.2443×10^5 cm^{-1} and the lattice constant was 2.866 Å.

4. Data omitted for clarity

Figure 3e of the main text presents data taken from samples with a total Fe thickness of 16 nm. In addition, there are data taken from samples with slightly different thickness. The relative drop in intensity of these data are corrected to account for the difference in excitation density (see methods). The trend observed in the figure's inset is then taken from the relative drop in each sample. However, the correction applied does not affect the time scale of the initial drop, which also depends on excitation density. Data from a $d_{Fe} = 12$ nm sample were omitted from Fig. 3a because the sub-ps time scale was notably longer than in other samples, while the amplitude was still corrected and presented in the inset. Presenting these data with the others could have therefore been misleading. Fig. S3 present the data from this sample.

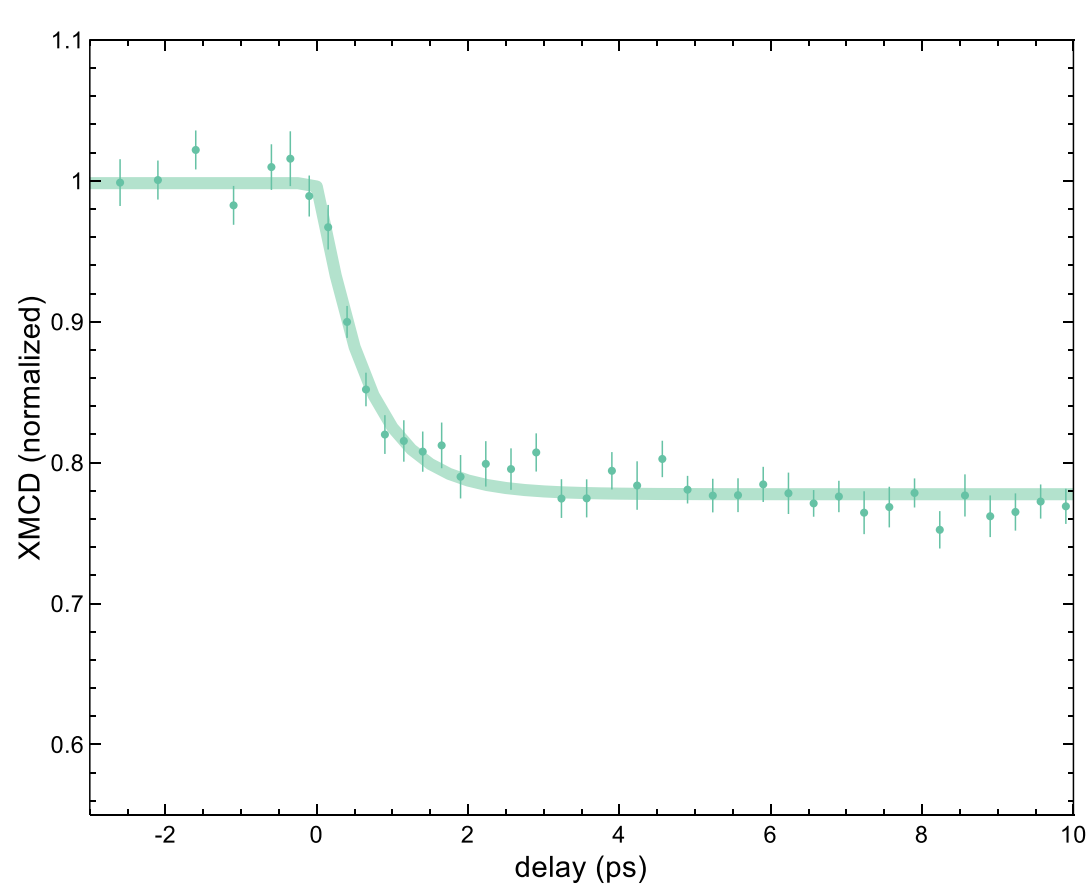

Fig. S3 – pump-probe XMCD data taken in transmission geometry taken from a $d_{Fe} = 12$ nm sample.

5. Phenomenological Simulations

This section details the simulation work done to reproduce the experimental results in the main text. We simulated the excitation of two slabs of Fe with different thicknesses (2 nm and 16 nm), during which both slabs experience the same excitation density. The simulation scheme is based on a phenomenological model, as sketched in Fig. S4a, with two steps.

Two-Temperature Model (TTM)

The first step is a two-temperature model (TTM) simulation of a single slab of Fe, in which the film thickness is not a parameter. The TTM produces a simulation of the temporal of the electron and lattice temperatures (T_e and T_L). This is expressed as:

$$\begin{aligned} C_e \frac{\partial T_e}{\partial t} &= -g_{ep}(T_e - T_L) + \kappa_e \nabla^2 T_e + P_{laser} \\ C_L \frac{\partial T_L}{\partial t} &= g_{ep}(T_e - T_L) + \kappa_L \nabla^2 T_L \end{aligned} \tag{1}$$

Here the heat capacities are $C_L = 3.667 \times 10^6\ J/m^3K$ (phonons) and $C_e = \gamma_{Fe} T_e$ with $\gamma_{Fe} = 764\ J/m^3K^2$ (electrons)[7]. The laser pulse is modelled as a gaussian of 29 fs FWHM with a total area of $|P_{laser}| = 45 \times 10^{21}\ \ W/m^3$. The electron-phonon coupling coefficient is $g_{ep} = 2 \times 10^{18}\ W/m^3K$ and the thermal conductance is $\kappa_e = 78\ W/mK$ (electrons) and $\kappa_L = 2\ W/mK$.

Fig. S4b (inset) presents the transient evolution of the T_{el} and T_{ph}, as simulated by the TTM. The time scales on which they evolve agree with the data in Fig. 2 and Fig. 3d of the main text, indicating a satisfactory choice of input parameters.

Atomistic Spin Dynamics Simulations

The second step is the atomistic spin dynamics (ASD) simulations, which receive $T_{el}(t)$ as input from the TTM. The ASD simulations receive the same input from the TTM for both slab thicknesses used (2 nm and 16 nm), reflecting identical excitation densities. The two ASD simulations differ only in the Fe thickness.

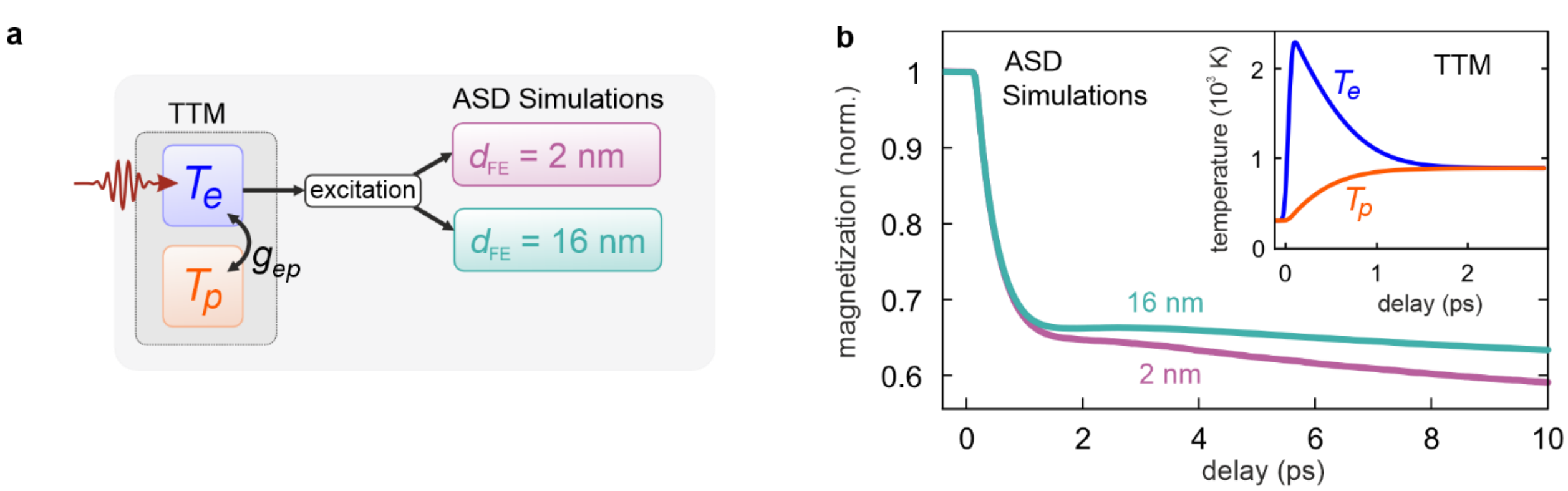

Fig. S4: Atomistic Spin Dynamics (ASD) Simulation. a sketch of the simulation workflow: a two-temperature model (TTM) is used as input for two different ASD simulations (ensuring comparable excitation density), that differ only in the thickness of the Fe slab. **b** Results of the simulations. The main panel depicts the Fe magnetization as a function of delay, as predicted by the ASD simulations. A larger demagnetization amplitude is predicted for a 2 nm Fe slab compared to a 16 nm slab, in qualitative agreement with the experiment. The inset presents the TTM results as functions of delay.

We simulate films as a varying number of monolayers with open boundary conditions in the film normal direction z to include interfaces and periodic boundary conditions in plane. Each spin is modeled in a semi-classical approach in the form of magnetic moments of constant magnitude, $\boldsymbol{\mu}_i = \mu_{Fe}\boldsymbol{S}_i$, where $\boldsymbol{S}_i$ is a unit vector. The magnetic system is described using a Heisenberg-type Hamiltonian of the form:

$$\mathcal{H}_i = -J\sum_{i,j=1}^{N} \boldsymbol{S}_i \cdot \boldsymbol{S}_j - d_4 \sum_{i=1}^{N} \left[S_{i,x}^4 + S_{i,y}^4 + S_{i,z}^4\right] \tag{2}$$

Here $J = 43.75\, meV$ is an effective isotropic exchange interaction, and $d_4 = 3.453\, \mu eV$ is the cubic magnetocrystalline anisotropy[8]. For the time evolution of the spins we employ the stochastic Landau-Lifshitz-Gilbert equation[9]

$$\frac{\partial \boldsymbol{S}_i}{\partial t} = -\frac{\gamma}{\mu_s(1+\alpha^2)}\left[\boldsymbol{S}_i \times (\mathbf{H}_i + \boldsymbol{\zeta}_i) + \alpha \boldsymbol{S}_i \times \left(\boldsymbol{S}_i \times (\mathbf{H}_i + \boldsymbol{\zeta}_i)\right)\right], \tag{3}$$

in which $\gamma \approx$ 176 GHz/T is the gyromagnetic ratio and $\alpha = 0.002$[10] is the Gilbert damping constant. The single cross product on the right-hand side describes the spins' precession around the effective field $\mathbf{H}_i$, with an additional stochastic contribution $\boldsymbol{\zeta}_i$ (see following). The double cross product leads to a relaxation of the spins towards the direction of the effective field.

The contribution ζ_i is the key contribution for this work, as it allows simulating the spins at finite temperatures. It behaves as gaussian white noise affected by the electron temperature, fulfilling:

$$\begin{gathered}\langle \boldsymbol{\zeta}_i(t)\rangle = 0 \\ \langle \zeta_i^\beta(t)\zeta_j^\xi(t')\rangle = \frac{2\mu_i^s \alpha k_B}{\gamma_i} T_e(t)\delta_{ij}\delta_{\beta\xi}\delta(t-t')\end{gathered} \tag{4}$$

This noise is uncorrelated in space and time, with β and $\xi \in \{x, y, z\}$. It depicts thermal fluctuations of the spins due to their coupling to a heat bath at temperature $k_B T$. This set of differential equations is then solved using Heun's method via a code developed in C++ and CUDA to simulate our system on GPUs for maximal efficiency. We simulate system sizes of 256×256×(number of layers). The simulations were performed on the HPC cluster SCCKN of University of Konstanz.

The demagnetization curves produced by the ASD simulations are presented in Fig. S4b. The ASD results are in qualitative agreement with our experiment: both ASD curves exhibit a primary sub-ps drop and a secondary few-ps drop, in agreement with Fig. 1e of the main text. Crucially, the drop in amplitude of the 2 nm slab is higher than the 16 nm slab's. As such, these simulations support the conclusion that the effect is magnetic and that it is not phonon-driven, but they fail to quantitatively reproduce the magnetization dynamics.

6. References

1. Kharoo, H. L., Gupta, O. P. & Hemkar, M. P. Temperature Dependence of Debye-Waller Factors in bcc Metals. I. *https://doi.org/10.1143/JPSJ.43.2030* **43**, 2030–2035 (2013).

2. Singh, V. P. & Hemkar, M. P. Anharmonicity and the Debye-Waller factors in BCC metals. *Journal of Physics F: Metal Physics* **7**, 753 (1977).

3. Chase, T. *et al.* Ultrafast electron diffraction from non-equilibrium phonons in femtosecond laser heated Au films. *Appl Phys Lett* **108**, 41909 (2016).

4. Maldonado, P. *et al.* Tracking the ultrafast nonequilibrium energy flow between electronic and lattice degrees of freedom in crystalline nickel. *Phys. Rev. B* **101**, 100302 (2020).

5. Waldecker, L. *et al.* Coherent and incoherent structural dynamics in laser-excited antimony. *Phys Rev B* **95**, 054302 (2017).

6. Maldonado, P., Carva, K., Flammer, M. & Oppeneer, P. M. Theory of out-of-equilibrium ultrafast relaxation dynamics in metals. *Phys Rev B* **96**, 174439 (2017).

7. Kittel C. Introduction to Solid State Physics, 8th edition, Berkeley. *John Wiley & Sons, New York* (1996).

8. Graham, C. D. Magnetocrystalline Anisotropy Constants of Iron at Room Temperature and Below. *Physical Review* **112**, 1117 (1958).

9. Brown, W. F. Thermal Fluctuations of a Single-Domain Particle. *Physical Review* **130**, 1677 (1963).

10. Barati, E., Cinal, M., Edwards, D. M. & Umerski, A. Calculation of Gilbert damping in ferromagnetic films. *EPJ Web Conf* **4**, (2013).
